# Acute intracerebral treatment with amyloid-beta (1–42) alters the profile of neuronal oscillations that accompany LTP induction and results in impaired LTP in freely behaving rats

**DOI:** 10.3389/fnbeh.2015.00103

**Published:** 2015-05-06

**Authors:** Alexander Nikolai Kalweit, Honghong Yang, Jens Colitti-Klausnitzer, Livia Fülöp, Zsolt Bozsó, Botond Penke, Denise Manahan-Vaughan

**Affiliations:** ^1^Medical Faculty, Department of Neurophysiology, Ruhr University BochumBochum, Germany; ^2^International Graduate School of Neuroscience, Ruhr University BochumBochum, Germany; ^3^Department of Medical Chemistry, University of SzegedSzeged, Hungary

**Keywords:** amyloid beta, Alzheimer’s disease, oscillations, cognitive deficits, hippocampus

## Abstract

Accumulation of amyloid plaques comprises one of the major hallmarks of Alzheimer’s disease (AD). In rodents, acute treatment with amyloid-beta (Aβ; 1–42) elicits immediate debilitating effects on hippocampal long-term potentiation (LTP). Whereas LTP contributes to synaptic information storage, information is transferred across neurons by means of neuronal oscillations. Furthermore, changes in theta-gamma oscillations, that appear during high-frequency stimulation (HFS) to induce LTP, predict whether successful LTP will occur. Here, we explored if intra-cerebral treatment with Aβ(1–42), that prevents LTP, also results in alterations of hippocampal oscillations that occur during HFS of the perforant path-dentate gyrus synapse in 6-month-old behaving rats. HFS resulted in LTP that lasted for over 24 h. In Aβ-treated animals, LTP was significantly prevented. During HFS, spectral power for oscillations below 100 Hz (δ, θ, α, β and γ) was significantly higher in Aβ-treated animals compared to controls. In addition, the trough-to-peak amplitudes of theta and gamma cycles were higher during HFS in Aβ-treated animals. We also observed a lower amount of envelope-to-signal correlations during HFS in Aβ-treated animals. Overall, the characteristic profile of theta-gamma oscillations that accompany successful LTP induction was disrupted. These data indicate that alterations in network oscillations accompany Aβ-effects on hippocampal LTP. This may comprise an underlying mechanism through which disturbances in synaptic information storage and hippocampus-dependent memory occurs in AD.

## Introduction

Information processing and storage in the hippocampus are enabled by phenomena such as synaptic plasticity and neuronal oscillations. Synaptic plasticity, in the form of long-term potentiation (LTP) and long-term depression (LTD), comprise the cellular basis for learning and memory in the hippocampus (Kemp and Manahan-Vaughan, 2007). Neuronal oscillations reflect ongoing processes within neuronal populations (Vanderwolf, 1969; Kramis et al., 1975; Bland, 1986; Dragoi and Buzsáki, 2006). In the hippocampus, theta (4–10 Hz) and gamma (30–100 Hz) oscillations are believed to reflect information processing associated with learning (Bland, 1986; Lopes da Silva et al., 1990; Stewart and Fox, 1990; Vertes and Kocsis, 1997; Buzsáki and Draguhn, 2004). Delta and alpha oscillations arise from the thalamus (Hughes and Crunelli, 2005; Zhang et al., 2012) and are forwarded to the hippocampus. Theta oscillations show the highest power levels in the hippocampus and originate from distinct sources, such as the medial septum/diagonal band of Broca (Lubenov and Siapas, 2009). Theta oscillations are associated with the appearance of gamma oscillations because of the physiological connection between gamma-inducing parvalbumin-positive interneurons (Fuchs et al., 2007) and theta oscillation-expressing pyramidal cells (Lubenov and Siapas, 2009). If gamma power is high, theta power usually becomes less, resulting from shunting inhibition of gamma oscillations (Vida et al., 2006).

Gamma oscillations are regarded as reflecting high-order synchronization between distinct brain areas. They have been suggested to be important for temporal encoding (Buzsáki and Chrobak, 1995), sensory binding of features (Gray et al., 1989) and in encoding and decoding of information (Lisman and Idiart, 1995; Lisman, 2005). Frequencies in the gamma range can be considered a synchronization gate, through which distinct brain areas are connected, and thereby share information over long distances between brain areas. This binding mechanism by frequency synchronization appears especially at the cortical level (Canolty et al., 2006). Hippocampal neuronal oscillations, particularly those in the theta-gamma frequency range, may serve to identify cell-assemblies that contribute to the experience-dependent processing and storage of information in the cortex and hippocampus (Lisman, 2005; Battaglia et al., 2011).

Strikingly, specific and characteristic patterns of theta-gamma activity occur during, and immediately, after tetanic afferent stimulation to elicit hippocampal LTP *in vivo*. These patterns are significantly different to theta-gamma activity that occur when the same stimulation protocol results in short-term potentiation (STP) or failure of potentiation (Bikbaev and Manahan-Vaughan, 2007). The specificity of the theta-gamma characteristics can serve as a predictor of the outcome of an attempt to induce LTP by means of high-frequency stimulation (HFS; Bikbaev and Manahan-Vaughan, 2008). This suggests in turn that these forms of neuronal oscillations are intrinsically associated with plasticity events in the hippocampus.

Alzheimer’s disease (AD) is accompanied by neuronal loss in a variety of brain regions such as the hippocampus (Cairns et al., 1991), amygdala (Tsuchiya and Kosaka, 1990) and cerebral cortex (Ogomori et al., 1989). The amyloid cascade hypothesis postulates that amyloid-beta (Aβ)-deposition is triggered mainly by Aβ(1–42) and that this aberrant aggregation behavior leads to neuronal death (Lambert et al., 1998; Barghorn et al., 2005; Karran et al., 2011) and cognitive dysfunction (Cleary et al., 2005; Geng et al., 2010). Aβ(1–42) aggregates to form soluble oligomeric species that are neurotoxic (Small and McLean, 1999). Low levels of pathogenic Aβ(1–42) accumulation induce synaptic dysfunctions long before synapse loss occurs (Selkoe, 2002). Furthermore, Aβ-plaques do not necessary precede the occurrence of cognitive alterations in the hippocampus: APP-PS1 transgenic mice that overexpress Aβ(1–42) exhibit cognitive deficits in an associative learning task at up to 12-months of age, first exhibited Aβ-deposits at the age of 18 months (Gruart et al., 2008). Thus, it may be the Aβ(1–42) peptide itself, rather than amyloid plaques that mediates hippocampal pathology and cognitive decline.

In line with this, treatment of rodent hippocampal slices with oligomeric Aβ(1–42), or intracerebral application *in vivo*, prevents LTP (Cullen et al., 1997; Chen et al., 2000; Walsh et al., 2002; Townsend et al., 2006; Lyons et al., 2007; Srivareerat et al., 2009). Whether theta-gamma oscillations, that occur in close association with information encoding through LTP, are also affected by Aβ(1–42)-neurotoxicity is unclear. Findings from related studies suggest, however, that neuronal oscillations are altered by Aβ(1–42) (Adaya-Villanueva et al., 2010), and increased power levels of hippocampal delta oscillations, accompanied by decreased theta power, occur following treatment of middle-aged rats with a secreted form of the amyloid precursor protein (sAPP; Sánchez-Alavez et al., 2007).

A tight correlation exists between hippocampus-dependent spatial learning, LTP and theta-gamma oscillations in the hippocampus (Bikbaev and Manahan-Vaughan, 2008; Habib et al., 2013). Thus, it may be the case that prevention of LTP, as a result of Aβ-treatment, may be associated with changes in theta-gamma oscillations in the hippocampus. The dentate gyrus is believed to be involved in pattern separation (Dees and Kesner, 2013) and context-reset in the hippocampus (Cheng, 2013), and is therefore a crucial hippocampal structure in terms of memory processing. The property of pattern separation is likely to be enabled by sparse encoding of information in the dentate gyrus, that may in turn be supported by theta-gamma modulated synaptic currents in this structure (Pernía-Andrade and Jonas, 2014). Along with LTP in the CA1 region (Hardy and Selkoe, 2002), LTP in the DG is impaired by treatment with Aβ(1–42) (Wang et al., 2002, 2009; Babri et al., 2012). To examine whether Aβ(1–42) affects neuronal oscillations that occur in association with the induction of hippocampal LTP, we assessed effects of acute intra-cerebral oligomeric Aβ(1–42) treatment on LTP and neuronal oscillations (at frequencies less than 100 Hz) in the DG of 6-month-old rats. We observed that Aβ(1–42) causes acute deficits in DG LTP along with alterations in hippocampal network oscillations. These data suggest that the impairment of hippocampal LTP that is mediated by Aβ(1–42) may result from disruptions in neuronal oscillations that are necessary for synaptic information processing.

## Materials and Methods

### Animals

The study was carried out in accordance with the European Communities Council Directive of 22 September, 2010 (2010/63/EU) for care of laboratory animals and after approval of the local ethic committee (Bezirksamt, Arnsberg). All efforts were made to minimize the number of animals used.

Male Wistar rats (6 months old at the time of surgery, Charles River, Germany) were used in all of the experiments. Animals were housed in a temperature-and humidity-controlled Scantainer with a constant 12-h light/dark cycle (lights on from 7 a.m. to 7 p.m.) where they had access to food and water *ad libitum*. In total 17 animals were used in this study.

### Surgery

Animals were anesthetized with sodium pentobarbital (52 mg/kg, intraperitoneally, i.p.) and underwent stereotaxic chronic implantation of electrodes and cannula in the right hemisphere, as described previously (Bikbaev and Manahan-Vaughan, 2007). In summary, a monopolar recording electrode was implanted in the granule cell layer of dentate gyrus (3.1 mm posterior to bregma, 1.9 mm lateral to the midline) and a bipolar stimulation electrode in the medial perforant pathway (6.9 mm posterior to bregma, 4.1 mm lateral to the midline). A guide cannula was inserted in the ipsilateral cerebral ventricle (0.5 mm posterior to bregma, 1.6 mm lateral to midline, 5.6 mm depth from skull surface) to enable subsequent injections via the intracerebral ventricle (i.c.v.). On the contralateral side, two anchor screws attached with stainless steel wires were inserted to serve as reference and ground electrodes. Test-pulse recordings during surgery aided depth adjustment of the electrodes. The electrodes were fixed to plastic sockets and the whole assembly was stabilized on the skull using dental cement. After surgery, animals were housed individually until they were 6-months-old, at which time the Aβ experiments were conducted.

At the end of the study, brains were removed for histological verification of electrode and cannula localization. Brain sections (16 μm) were embedded in paraffin, stained according to the Nissl method, using 1% toluidine blue, and then examined using a light microscope, as described previously (Hansen and Manahan-Vaughan, 2014). Data from brains in which incorrect electrode localization was found were excluded from the study.

### Measurement of Evoked Potentials

One day before the experiments started, animals were transferred to the experiment room and placed in the recording chambers so that we could ensure that full habituation to the environment had occurred before experiment were begun. The electrophysiological recordings were performed in 40 (L) × 40 (W) × 40 (H) cm lidless recording chambers wherein the rats could move freely and had access to food and water *ad libitum*. Intrahippocampal EEG was recorded concurrently with recordings of evoked potentials.

For recordings of evoked potentials, the perforant path was stimulated with test-pulses (0.025 Hz) using single biphasic square wave pulses, of 0.2 ms duration. Both the population spike (PS) and the field excitatory postsynaptic potential (fEPSP) were assessed. The fEPSP was measured as the maximal slope through the five steepest points obtained on the positive deflection of the potential. PS amplitude was measured as the amplitude through the five steepest points obtained on the first negative deflection of the PS.

Prior to each plasticity experiment an input-output (i/o) curve was determined, whereby the afferent stimulation intensity was increased in steps of 100 μA from 100 μA up to 900 μA. The intensity that generated 40% of the maximum responses was used for all subsequent experiments. For each time-point measured during the experiments, five records of evoked responses were averaged. The first 6 time-points (30 min) recorded at 5 min intervals were used as a baseline reference, and potentials evoked at all subsequent time-points are shown in relation to the average of these 6 points (for time-points used for EEG analysis, see section on network analysis below) intracerebroventricular (i.c.v.) injections were given after the 30 min baseline recording period via the implanted guide cannula. A further 30 min of baseline recording was conducted after the injection, after which HFS to elicit LTP was applied. HFS comprised 15 pulses at 200 Hz repeated 10 times with a 10 s interval. Immediately prior to, during and after HFS we verified that the animals were stationary, resting and had their eyes open. By this means we could assume that their behavioral states were as equivalent as possible.

We previously observed that changes in the spectral power of theta and gamma oscillations predicts for the success of HFS in inducing LTP (Bikbaev and Manahan-Vaughan, 2007, 2008). In order to assess the longevity of synaptic potentiation elicited by HFS, we monitored evoked responses for 25 h. Thus, evoked potentials were recorded for 4 h after HFS and for one additional hour 24–25 h after HFS. The first 3 time points after HFS were recorded at 5 min intervals, following by recordings at 15 min intervals. HFS comprised 15 pulses at 200 Hz repeated 10 times with a 10 s interval. This protocol is effective in inducing LTP (>24 h) in the dentate gyrus *in vivo* (Naie and Manahan-Vaughan, 2004; Kemp and Manahan-Vaughan, 2008). Exceptions occur, however, whereby some animals do not respond to HFS with LTP (Bikbaev and Manahan-Vaughan, 2007, 2008). Animals were thus screened for successful LTP prior to commencing the study. Those that did not show robust LTP (>24 h) were excluded from subsequent experiments. This screening was conducted at least 7 days before starting the Aβ experiments, at which point each animal was screened to verify that i/o responses were equivalent to those obtained prior to the control LTP experience, i.e., the control LTP was no longer evident at the time of commencing Aβ experiments.

### Analysis of Network Activity

Our network analysis focused on the changes in spectral power of neuronal oscillations that occur during, and immediately after HFS, as we had previously observed a tight correlation between the changes spectral power of theta and gamma oscillations during and immediately after HFS, and the success of HFS in inducing LTP (Bikbaev and Manahan-Vaughan, 2007, 2008). Thus, although we monitored evoked potentials for 25 h after HFS to verify that our control animals expressed LTP that lasted for over 24 h (as opposed to STP), we restricted our network activity analysis to the time period immediately associated with HFS (Figures 2A–D).

**Figure 1:**
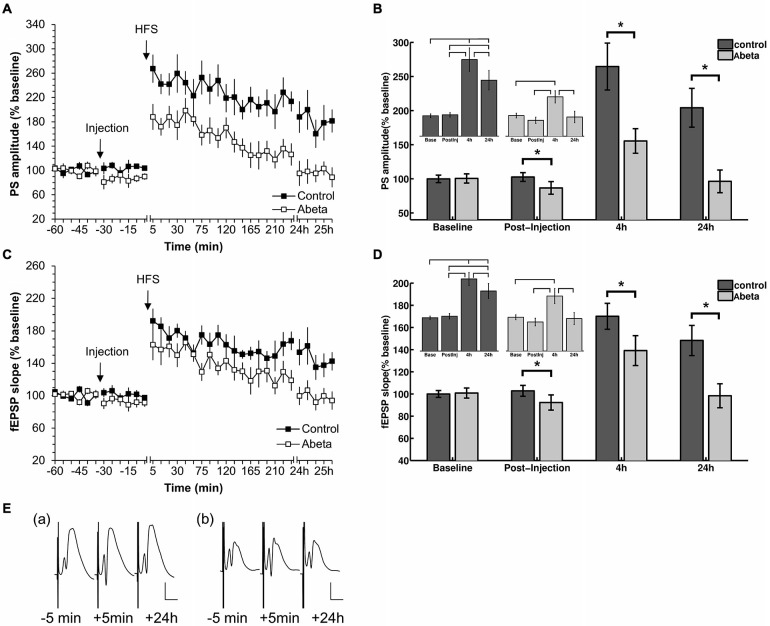
**High-frequency stimulation (HFS) elicits robust LTP that persists for over 24 h in vehicle-treated adult rats but not in Aβ-treated rats. (A,C)** Acute injection of soluble Aβ oligomers resulted in impaired LTP in the dentate gyrus *in vivo* with regard to both population spike (PS) **(A)** and field excitatory postsynaptic potential (fEPSP) **(C). (B,D)** Figures show bar-charts representing pooled PS **(B)** and fEPSP **(D)** values from the graph shown in **(A)**. The data summarize mean values during 6 time-points recorded pre-injection (baseline) and the 6 time-points after injection but prior to HFS (post-injection), as well as the mean values obtained 4 h and 24 h after HFS. The smaller bar-chart insets in each graph show the results of within-group comparisons. Significant within-group comparisons are indicated by lines drawn in the insets within parts **(B,D)**. Significant between-group responses are marked with an asterisk (*) in **(B,D). (C)** Between-group comparison of PS amplitudes **(C)** revealed significantly lower values for Aβ-treated animals in the post injection time-period, as well as 4 h and 24 h after HFS. Within group comparisons of PS amplitudes (inset in **C**) revealed significantly higher values 24 h after HFS compared to the pre-injection and post-injection phases, and post-injection only, for control animals. The same pattern can be seen for the fEPSP responses for between-group and within-group comparisons. Pre-HFS baseline responses baselines did not differ between groups for either fEPSP or PS. **(E)** Original analog traces show representative field potentials evoked 5 min before, 5 min after, and 24 h after HFS from: (a) a vehicle-treated rat; and (b) an Aβ-treated rat. Vertical scale bar corresponds to 5 mV; horizontal scale bar corresponds to 5 ms.

**Figure 2:**
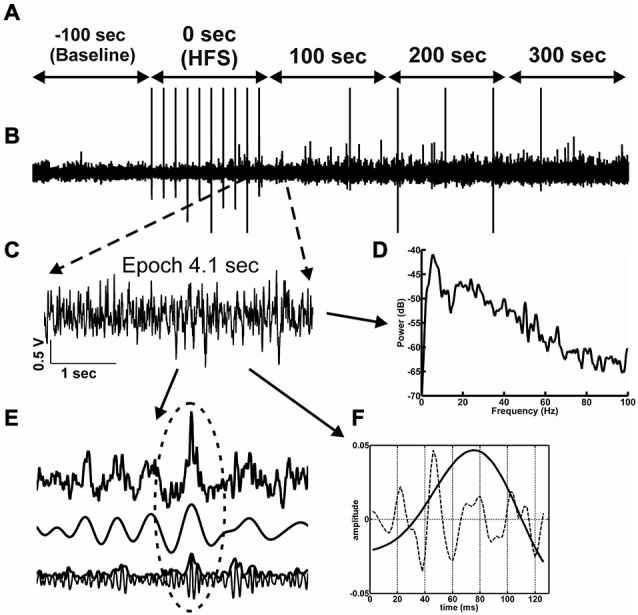
**Network activity and analysis approach. (A)** The time-line for the EEG analysis comprised 500 s of EEG recording. One hundred seconds of EEG (prior to HFS) are used as baseline (−100 s time-point). The time-point “0’ represents the period during which HFS was applied. The 3 subsequent time-windows reflect EEG recordings 100 s, 200 s and 300 s after HFS 300 s. **(B)** Raw EEG, in which stimulus artifacts induced by test-pulse afferent stimualtion or HFS can is indicated by vertical lines. **(C)** From the raw EEG, 10 artifact-free 4.1 s epochs **(C)** were extracted from every 100 s time window **(A). (D)** The raw EEG epochs were analyzed as to their relative change relative to baseline in power levels for the frequencies in the delta, theta, alpha, beta and gamma ranges. **(E)** The principal of inter-frequency phase-to-amplitude-coupling (PAC) detected by envelope-to-signal correlation (ESC) is shown in 1 s of EEG. The resulting correlation score for one *single* 4.1 s epoch is influenced by the appearance of envelope-to-signal correlations. This means the shape of the high frequency filtered signal-envelope (bottom signal in **F**) and the shape of the low frequency filtered signal (middle signal in **F**) are approximately the same. The upper signal in **(F)** shows the raw EEG signal. The region at the dotted ellipse in the signal shows a typical envelope-to-signal correlation: The shape of the envelope of the fast signal shows approximately the same shape as the amplitude of the compared slow signal. The more often and pronounced this phenomenon appears within an analyzed EEG epoch, the higher will be the overall PAC-ESC score for this epoch. **(F)** The cosine phases of theta oscillations (black line in **F**) and the gamma oscillations during these theta cycles (dotted line in **F**) were extracted by using the Hilbert transformer. These data were then used to determine the peak-to-slope amplitudes of theta cycles and the mean gamma amplitudes during these theta cycles.

The intra-hippocampal electroencephalogram (EEG) was obtained during recordings of evoked potentials from the dentate gyrus granule cell layer. EEG was sampled (1 kHz, gain 100×, 0.1 Hz–20 kHz) using Spike2 software (Cambridge Electronic Design, UK) and stored for subsequent offline analysis. For this purpose, the EEG signal was digitally down-sampled to 500 Hz, 250 Hz or 125 Hz depending on the band-pass filter applied. HFS generated large spike artifacts in the EEG of both vehicle-treated and Aβ-treated animals (Figure 2B). The interval between stimuli during HFS was 10 s, and 15 stimuli were given, thus, the HFS period spanned 90.7 s. To evaluate the effects of HFS on oscillatory activity in delta (2–4 Hz), theta (4–10 Hz), alpha (10–12 Hz) beta (12–28 Hz) and gamma (30–100 Hz) frequency ranges, 4.1 s-long artifact-free epochs of EEG were extracted from 10 s recording periods (Figure 2B) to enable comparison with artifact-free EEG activity in the periods between the HFS stimuli. Baseline data were obtained as 4.1 s-long artifact-free epochs in 10 time-intervals, within the 100 s of EEG that occurred immediately prior to HFS. These data were used as a reference for analysis of data obtained during or after HFS (i.e., taken as 100% for further normalization). In addition, similar epochs were assessed in a period of 100 s during HFS and a period of 300 s immediately after the conclusion of HFS. Subsequently, a set of digital finite impulse response filters (−3 dB points for band-stop: 48.5–55 Hz (notch-filter), band-pass: 2–4 Hz, 4.5–10 Hz, 10–12 Hz, 12.5–28 Hz, 29.5–99.5 Hz; transition gap 1, 1.5 and 2.5) was applied to the extracted epochs (Bikbaev and Manahan-Vaughan, 2007; Tsanov and Manahan-Vaughan, 2009). For all artifact-free epochs, fast Fourier analysis (FFT) with Hamming window function and 512, 1024 or 2048 frequency bins was performed depending on the band-pass filter and sampling rate used. The mean values for each mean square spectrum (MSS) were calculated by a self-written Spike2-script (containing the functions SetPower() and ChanMeasure()), and the results were stored in ascii-format. In addition, the raw wave forms for all artifact-free epochs were also stored in ascii-format for later analysis using MATLAB (MathWorks, Natick, MA, USA, version: R2012a).

The trough-to-peak amplitude of filtered theta cycles (Figure 2E) was calculated using the Hilbert transformer (Matlab-function: hilbert()) to create an analytic signal. The maximum elongation of each excised cosine phase cycle was measured, and by determining the length of each cosine phase cycle, the frequency was recalculated. The same procedure was applied for gamma cycle analysis during each theta phase cycle. The absolute values of spectral power, the amplitude of the theta cycle and the mean amplitude of the gamma oscillations per theta cycle in the artifact-free epochs were normalized for each individual animal to respective mean values for the 100 s-long baseline period, and the relative values were statistically analyzed. In addition, the amount and length of each cycle during each epoch was stored for further analysis.

Measurements of inter-frequency phase-to-amplitude coupling (PAC) were performed using a combination of published Matlab-functions (Onslow et al., 2011) and self-written Matlab-functions on stored artifact-free raw EEG in MATLAB. A high variety of technical approaches to quantify phase-to-amplitude coupling has been published, each with their own pros and cons, but we decided to use the envelope-to-signal correlation (ESC) rating (Bruns and Eckhorn, 2004) due to its reliability with regard to short signals, as these were the focus of our study (Figure 2E). To assess envelope-to-signal correlations, we compared the envelope of an oscillatory signal to the signal of another oscillator, which occurs at the same time. If the shape of the envelope of a signal and the shape of another signal are approximately the same, then the ESC score will be high. This is often the case if a beat in a fast oscillator occurs, and the envelope of this beat shows the same shape as the amplitude of a slower oscillator. We used the following settings for PAC-ESC measurements: Morlet wavelet filter width: 7, FFT-size: 200, shuffling windows: 200. Raw wave signal sampling rate: 500 Hz. If not otherwise mentioned, the default settings of the provided function were used. The mean PAC-ESC score for all epochs was acquired for all rats for each test group, specific to the time-windows assessed before, during and after HFS.

### Statistical Analysis

For LTP experiments, results were expressed as the mean percentage ± standard error of the mean (S.E.M.) of the average of the first 6 recordings. The whole statistical analysis was performed in “R” version 3.0.1 (The R Foundation of Statistical Computing) with in-built packages and “nortest”. The Anderson-Darling-test (ad.test()) was applied to assess for normal distribution. If the Anderson-Darling-test was significant, the non-parametric Wilcoxon-test (wilcox.test()), or a self-written R-function for the Kruskal-Wallis test, with Tukey *post hoc*-tests and Bonferroni correction was applied. Otherwise parametric statistical tests were applied. The effect of time included five levels for pre-HFS (100 s), during HFS (100 s) and post-HFS (300 s), as demonstrated in Figure 2A. The results of analysis were expressed as mean % pre-HFS values ± S.E.M. The global probability level interpreted as statistically significant was *p* < 0.05 (*). If Bonferroni correction was applied the threshold for the *p*-value was lowered depending on the amount of compared results, taking into account the multiple comparisons problem.

### Peptide Treatment

The oligomeric (1–42) peptide was synthesized in the following way: The isopeptide precursor iso-Aβ 42 was synthesized by Fmoc-chemistry and transformed at neutral pH to Aβ42 by O→N acyl migration in a short period of time, resulting in a water soluble oligomeric mixture of Aβ(1–42) oligomers. The aggregation grade of these oligomers, thus formed, could be better standardized. The synthesis and characterization of the aggregation process of Aβ(1–42) was conducted as previously described (Bozsó et al., 2010). In this study, the soluble Aβ(1–42) oligomeric peptide was prepared by incubating the the oligomeric “iso-Aβ 42” peptide in PBS at pH 7.4 for 3 h at a concentration of 50 μM. It was subsequently diluted to the final concentration of 10 μM, shock-frozen with liquid nitrogen and stored at −80°C. The Aβ solution was thawed (at room temperature) shortly before intra-cerebral injection using an ultrasonic device for 5 min. The concentration of Abeta was chosen based on reports of others as to its efficacy in preventing CA1 LTP *in vivo* (Klyubin et al., 2004). Five minutes prior to commencing injections, the injection cannula was inserted into the guide cannula. The total volume injected was 5 μl and this was injected at a rate of 5 μl/min. Five minutes after conclusion of the injection, the cannula was carefully withdrawn from the guide cannula. No differences in animals’ behavior were detected after Aβ(1–42)-injection compared to controls.

## Results

### Acute Injection of Aβ Impairs LTP in the Dentate Gyrus of Adult Rats

In this study, we first confirmed that all animals expressed robust LTP in response to HFS. At least 1 week later, after evoked potentials had returned to pre-HFS levels, these animals were randomly assigned into two groups. One group received acute i.c.v. injection of Aβ (*n* = 8) after 30 min of recording of basal synaptic transmission. The other group was treated with vehicle at the same time-point (*n* = 9). Thirty minutes after the acute injection, HFS (200 Hz) was applied that resulted in robust LTP that persisted for over 24 h in control animals (Figures 1A,B). In contrast, acute injection of Aβ caused a significant impairment in LTP compared to controls. Between-group statistical analysis (Figures 1B,D) revealed significant treatment effects between the two groups with regard to both PS (Figures 1A,B) and fEPSP (Figures 1C,D). Significantly lower values occurred at the post-injection time point for Aβ-treated animals compared to controls (Figures 1B,D). Furthermore, within-group analysis revealed that evoked responses in the Aβ-group after HFS-application were not significantly different from the baseline values of the Aβ-group. (PS: ${\text{χ}}_{\left(\text{3}\right)}^{\text{2}}$ = 93.6, *p* < 0.05; Bonferroni corrected Tukey-based *post hoc* test: Base/Post-injection: *p* > 0.083; fEPSP: ${\text{χ}}_{\left(\text{3}\right)}^{\text{2}}$ = 93.6, *p* < 0.05; *post hoc* test: Base/Post-injection: *p* > 0.083). Between group comparisons revealed that the pre-HFS baseline responses for control and Aβ-group did not significantly differ (PS: *t*-test: *t*_(98)_ = −0.13, *p* > 0.05; fEPSP: (Wilcoxon-test: *W* = 1204.5, *p* > 0.05).

Our finding that dentate gyrus LTP is impaired LTP following acute treatment with oligomeric Aβ(1–42) *in vivo*, is in line with reports of others, that Aβ(1–42) impairs CA1 LTP both *in vitro* and *in vivo* (Cullen et al., 1997; Chen et al., 2000; Walsh et al., 2002; Lyons et al., 2007; Srivareerat et al., 2009).

### Aβ-Injection Disrupts the Typical Pattern of Hippocampal Network Activity During HFS

**Figure 3:**
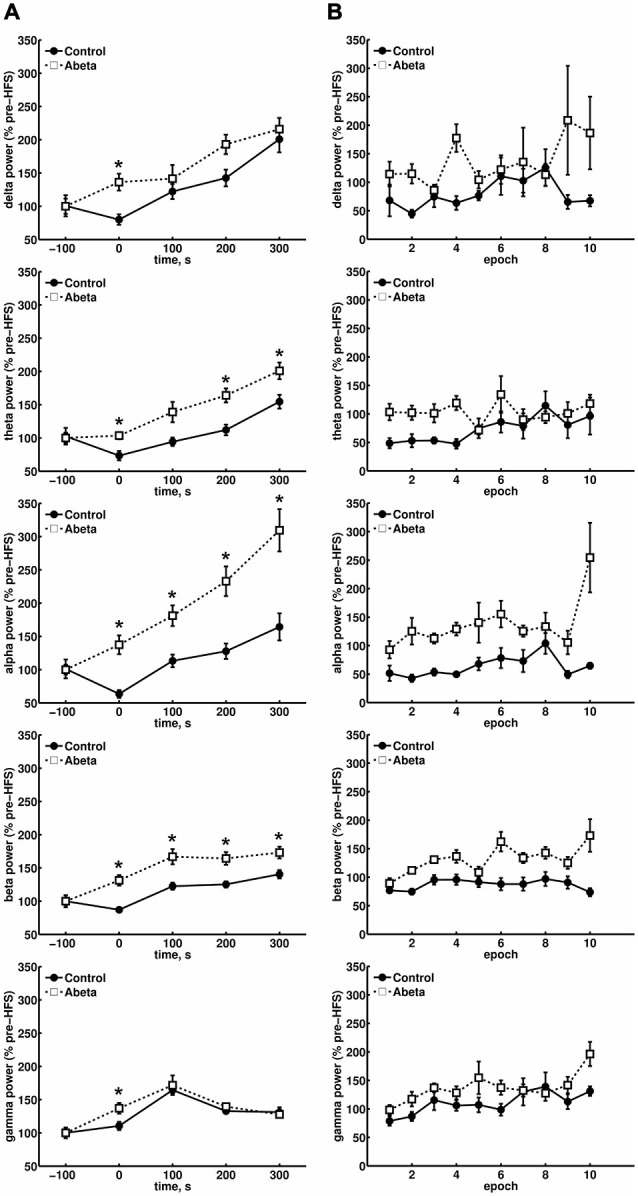
**Overview of relative spectral power for oscillations below 100 Hz for control and Aβ-treated animals. (A)** Relative changes in spectral power for delta, theta, alpha and beta bands in control and Aβ-group before, during and 300 s after HFS comprising 500 s EEG analysis are shown. Each time-point contains the mean values of 10 epochs from 4.1 contiguous seconds of EEG data derived from spectral power analysis of the respective, pre-filtered frequency band. The power levels of the baselines for the respective frequencies did not differ significantly. The relative spectral power for all frequency bands in Aβ-treated animals showed significantly higher scores during HFS compared to controls (Wilcoxon-test: *p < 0.05). In addition, the relative theta, alpha and beta power also showed significantly higher relative power levels for the Aβ-group compared to controls. **(B)** The change in relative power levels for single epochs during HFS induction are shown, and demonstrate that a suppression of theta power at the beginning of HFS can be seen in controls that is followed by a gradual increase back to baseline values. This response profile is absent in the Aβ-group. Only control animals exhibit a suppression of the relative network power levels of all frequency bands at the beginning of HFS application.

Continuous raw EEG recordings are more sensitive to noise disruptions than recordings of evoked fEPSPs or PS. After excluding recordings that exhibited severe artifacts in the EEG, we analyzed data from 6 vehicle-treated rats (control group) and 5 Aβ-treated animals (Aβ-group). Baseline EEG was equivalent in these groups: the Wilcoxon sign-rank test revealed no significant difference in the baseline EEG of the control and Aβ-group, with regard to all frequency bands assessed (Delta: *W* = 1356, *Z* = 0.582, *p* > 0.05, *r* = 0.05; Theta: *W* = 1241, *Z* = 1, *p* > 0.05, *r* = 0.09; Alpha: *W* = 1362, *Z* = 0.69, *p* > 0.05, *r* = 0.06; Beta: *W* = 1551, *Z* = −0.3, *p* > 0.05, *r* = −0.02; Gamma: *W* = 1482, *Z* = 0.11, *p* > 0.05, *r* = 0.01). In contrast, during HFS, the relative power levels of all frequencies assessed (δ, θ, α, β, γ) were significantly lower in control animals compared to Aβ-treated animals (Figure 3A) (Wilcoxon-test: *p < 0.05). This significant alteration in relative EEG power may reflect a general network effect induced by the Aβ-treatment. A closer examination of the relative power levels during HFS (Figure 3B) revealed that the relative theta power levels of the control group were suppressed to 50% of baseline power levels during the initial half of the HFS period (Figure 3B, second graph from top) (Wilcoxon-test: *p* < 0.05), but then continuously rose in the second half of HFS. This aligns with reports as to the typical response of theta power levels to HFS application in healthy animals (Bikbaev and Manahan-Vaughan, 2007, 2008). For the Aβ-treated animals there was no such effect on the theta power levels, rather theta power steadily increased in the period after HFS. Approximately the same pattern (of a steady power increase) can be observed for the relative delta (Figure 3B, first graph from top), and alpha power levels (Figure 3B, third graph from top). Strikingly, healthy animals did not exhibit this delta and alpha response pattern following HFS. In addition, the relative delta power levels measured in Aβ-treated animals appear to be less stable during HFS induction compared to vehicle-treated animals.

We considered whether the last data point during HFS, with regard to the relative alpha power levels in the Aβ-group, might be an outlyer (Figure 3B, third graph from top). However, if this effect was an artifact in the raw EEG, all other frequencies would have been affected in their relative power levels, and no such effect can be seen if one compares the behavior of the last data point during HFS induction for all other frequencies shown (Figure 3B). In controls, beta power (Figure 3B, fourth graph from top) was surprisingly stable in its suppression over time, during HFS. This response profile was absent in the Aβ-group. In controls, gamma power levels during HFS (Figure 3B, graph at bottom) showed the same relative behavior as could be observed for relative theta power levels (Figure 3B, second graph from top).

In Aβ-treated animals, this coupling of theta and gamma activity was absent. For example, during epoch 5 and 6 (Figure 3B, second graph from top), the relative theta power in Aβ-treated animals, remains unaffected by HFS. Nonetheless, the gamma power (Figure 3B, graph at bottom) rises during HFS. This finding suggests that an uncoupling of theta and gamma oscillations occurs on a functional level as a result of Aβ-treatment. The profile of change in relative gamma power in control animals that subsequently exhibit robust LTP fits to previous results (Bikbaev and Manahan-Vaughan, 2007). Thus, in controls, the relative gamma power levels (Figure 3A, graph at bottom) reach a maximum 100 s after HFS and thereafter decrease in relative power during the following 200 s. This pattern is expressed in both vehicle and Aβ-treated groups. However, during HFS, the relative gamma power in the Aβ-group is significantly higher compared to control animals (Figure 3A, graph at bottom).

The theta-gamma power patterns expressed by the Aβ-treated animals we observed as a consequence of HFS, were completely distinct from those patterns observed in healthy animals that expressed LTP, short-term plasticity or failure of LTP after HFS (Bikbaev and Manahan-Vaughan, 2007, 2008). As the EEG responses we observed for the Aβ-group animals do not fit to the typical patterns we could see in healthy animals for distinct types of plasticity expression, we conclude that these patterns reflect a pathological picture induced by Aβ-treatment.

### Theta and Gamma Amplitudes During HFS are Enhanced by Aβ-Treatment

**Figure 4:**
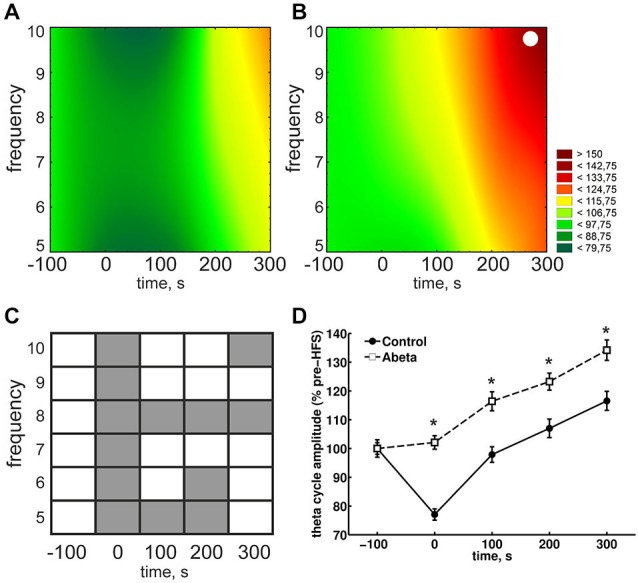
**Overview of relative theta amplitudes at frequencies from 5–10 Hz for control and Aβ-treated animals. (A,B)** The results for peak-to-slope amplitudes of cosine-phase theta cycles, normalized to baseline for control **(A)** and Aβ-treated animals **(B)** are shown. The color scale represents percent to the mean baseline values in **(A,B)**. The results for peak-to-slope amplitudes for control group **(A)** show that during HFS, a suppression of theta amplitudes for almost all displayed frequencies can be seen. This does not appear in Aβ-group **(B)**. However within-group statistical analysis revealed that the suppression in the control group is not significant for single theta cycle frequencies (White dots in **(A)** and **(B)** reflect significant different values compared to baseline according to Kruskal-Wallis-Test with Bonferroni corrected Tukey-based *post hoc* analysis for within group analysis). **(C,D)** The gray marks in the table **(C)** represent significant differences (according to the Bonferroni-corrected Wilcoxon test) in relative theta amplitude levels at different time-points and frequencies between control **(A)** and Aβ-treated animals **(B)**. White marks indicate an absence of significant difference. The color scale represents percent to the mean pre-HFS value in **(A,B)**. The suppression of theta cycle amplitudes during HFS of control, but not Aβ-treated animals animals is also expressed in the pooled frequencies **(D)**. Values in the graphs are expressed as the mean ± standard error of the mean (SEM).

Due to the physiological coupling of theta and gamma amplitudes and their importance for successful LTP induction, we took a closer look at the single frequencies of theta cycles and gamma amplitudes during these theta cycles. During HFS, theta amplitudes were significantly higher in the Aβ-group compared to the control group (Figures 4A,B, time point 0) for each frequency from 5 to 10 Hz. In controls, theta cycle amplitudes through HFS were significantly lower at all frequencies from 5 to 10 Hz. By contrast, the 8 Hz theta peak-to-slope amplitudes were significantly higher in the Aβ-treated animals from the time-point of HFS application onwards (Figure 4C). Thus, the main effect of Aβ-treatment seems to be in this 8 Hz frequency range. This lack of suppression of 8 Hz theta during and after HFS might have contributed to the significantly higher relative peak-to-slope theta values, which can be observed in the pooled data (Figure 4D).

### Mean Gamma Amplitudes Within a Theta Cycle are Altered Following Aβ-Treatment

**Figure 5:**
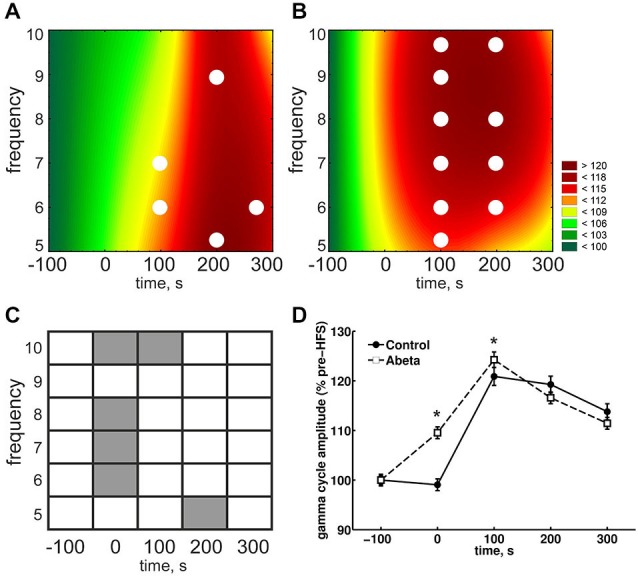
**Overview of relative gamma amplitudes during each theta cycle for control and Aβ-treated animals**. The mean gamma amplitudes during each cosine theta cycle (normalized to baseline) for control **(A)** and Aβ-treated animals **(B)** are shown. The color scale represents percent to the mean baseline values in A and B. The relative mean gamma amplitudes during each theta cycle are higher during HFS in Aβ-treated animals compared to control animals, as can be seen by a between-group comparison **(C)**. This significant difference is also reflected in the pooled data **(D)**. Both groups show the same pattern: The relative mean gamma amplitudes during each theta cycle increases till the time-point of 100 s (post-HFS) and then decays in amplitude during the following 200 s. All the same, the relative mean gamma amplitude at time point 100 s in the Aβ-group is significantly higher compared to controls. Within-group statistical analysis revealed that the relative mean gamma amplitudes are higher at all theta cycle frequencies at time-point 100 s for Aβ-treated animals (white marks in **B**) but not for controls (white marks in **A**). Within-group comparison for the pooled data **(C)** showed that the relative gamma amplitudes are higher compared to baseline only for Aβ-animals. White dots in **(A)** and **(B)** reflect significantly different values compared to baseline according to the Kruskal-Wallis-Test with Bonferroni corrected Tukey-based *post hoc* analysis.

Neuronal oscillations are influenced by interdependencies between the relative timing (phase) and power (amplitude) of rhythmic firing of neurons within neuronal networks (Onslow et al., 2011). These may serve to dynamically coordinate functionally related neuronal ensembles during behavior (Onslow et al., 2011). In addition to analysing theta and gamma activity as separate components of the EEG, we assessed the relative mean gamma amplitudes during each theta cycle (Figure 4). This type of cross-frequency coupling reflects phase synchrony, during which a certain number of higher frequency gamma cycles can occur within a single cycle of the lower frequency theta cycle (Tass et al., 1998). Gamma oscillations are mediated by parvalbumin-positive interneurons (Fuchs et al., 2007). A physiological relationship exists gamma and theta oscillations, which is based on the connection of theta expressing pyramidal cells and the gamma-inducing parvalbumin-positive interneurons. Thus, if relative slope-to-peak theta amplitudes are affected by Aβ-treatment, the mean gamma amplitudes during each theta cycle should also be affected. During HFS we did indeed observe a significant effect on relative mean gamma amplitudes during theta cycles compared to baseline levels (Figures 5A,B, time point 0) for either controls or Aβ-treated animals. However, at the time-point of HFS treatment, significantly higher mean gamma amplitudes occured in Aβ-treated animals (Figure 5C). This effect is also reflected in the pooled data (Figure 5D). The relative mean gamma amplitudes during each theta cycle reach their maximum at time-point 100 s (after commencement of HFS) for both groups. However, the mean gamma amplitudes at each theta cycle frequency at time point 100 s only differ significantly in the Aβ-group (Figure 5B). This indicates that the higher peak-to-slope theta amplitudes that occur in the in the Aβ-group (Figure 4) also affect the mean gamma amplitudes during each theta cycle. Thus, the direct physiological connection between theta and gamma oscillations on a cellular level may not be affected by Aβ. The increases in mean gamma amplitudes in the Aβ-treated animals may rather appear as a *side-effect*, whereas the main effect of Aβ-treatment is on the theta power levels.

### Envelope-to-Amplitude Coupling During HFS is Impaired by Aβ-Treatment

**Figure 6:**
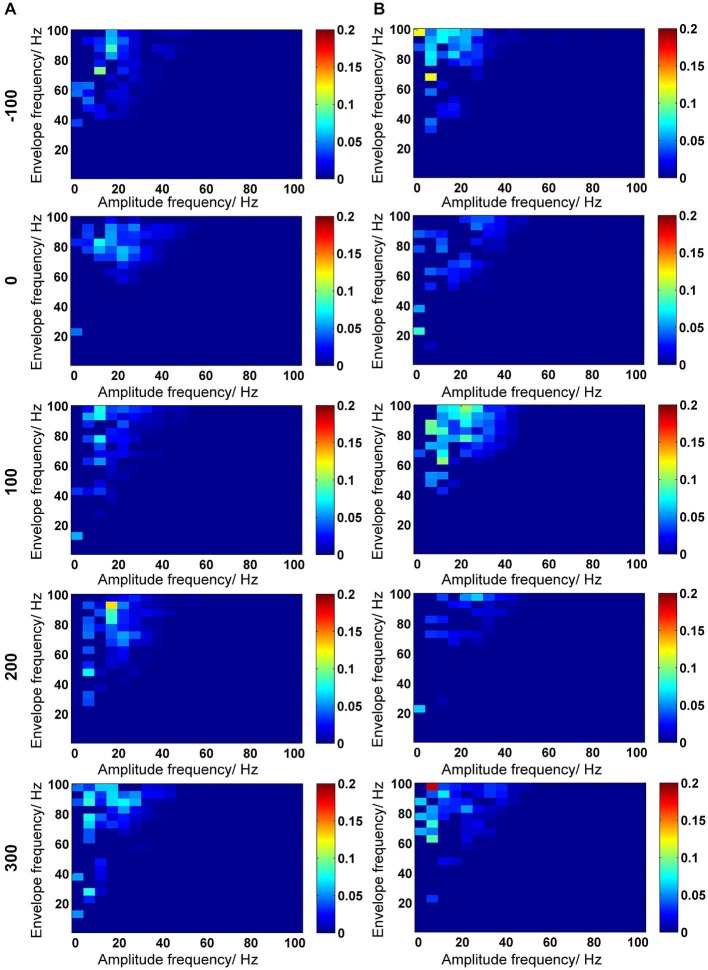
**Inter-frequency phase-to-amplitude coupling (PAC) quantified by envelope-to-signal correlation (ESC). (A,B)** PAC-ESC scores for control animals **(A)** and for Aβ-treated animals **(B)**. The time-line for EEG time-windows that were analyzed are shown at the left-side of the figure (see also Figure 2). The PAC-ESC scores were pooled from 10 measurements of 4.1 s epochs in 100 s time windows. The raw EEG was binned into center frequencies from 3–98 Hz ± 2 Hz resulting in 20 bins with 5 Hz frequency range. The *y*-axis reflects the envelopes of the binned signal whereas the *x*-axis reflects the frequencies of the signal amplitudes. The PAC-ESC scores, resulting from comparison of envelopes and amplitudes at binned frequencies, are expresses on the color scale for each plot. The probability that envelopes of a fast signals are fitting to the shape of amplitudes from slower signals is higher. For this reason, the highest PAC-ESC scores can be observed at the top left of each single plot. This relationship keeps stable for control and Aβ-animals for all time points **(A,B)**. Particularly the PAC-ESC scores for frequencies in the range of theta to beta, and high gamma oscillations are prominent during the whole 500 s of analyzed EEG. However from this analytical perspective no clear differences between controls and Aβ-treated animals can be observed.

**Figure 7:**
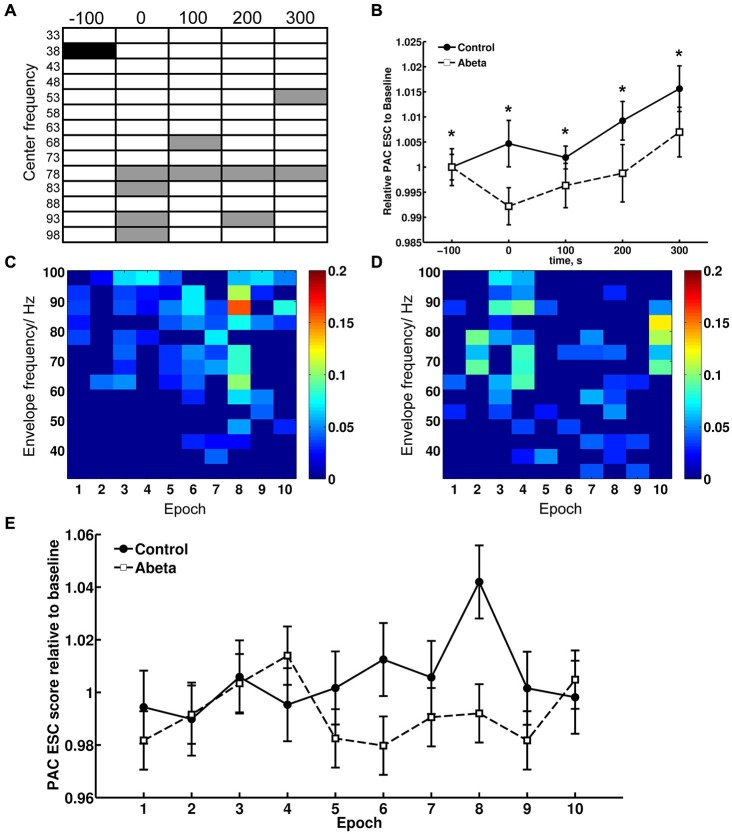
**Theta-gamma coupling estimated with PAC–ESC for control and Aβ-group**. To estimate the effects of Aβ-treatment on theta-gamma coupling in terms of envelope-to-signal correlations, the PAC-ESC scores in range of theta and gamma oscillation were extracted from the whole PAC-ESC analysis (see Figure 6). All plots in Figure 7 show coupling scores solely for theta and gamma oscillations. A within-group statistical comparison of normalized PAC-ESC scores from control group and Aβ-group animals **(B)**, revealed a significant effect over time on PAC-ESC scores compared to baseline only for Aβ-treated animals (Kruskal-Wallis test: *p* < 0.01). However a Bonferroni-corrected Tukey-based *post hoc* analysis revealed no significant PAC-ESC scores compared to baseline for both groups. A between-group comparison revealed significantly lower values Aβ-treated animals (Bonferroni-corrected Wilcoxon test: *p < 0.005) for all time-points. This observation on the pooled binned gamma envelopes is however undermined by the significant difference in baselines of control and Aβ-treated animals. Nonetheless, not all gamma center-frequencies show significant variations in baseline **(A)**. Black-marked center-frequencies in table **(A)** show significantly variant baselines. Gray-marked center-frequencies show significant differences at the respective center-frequencies between control and Aβ-animals (Bonferroni-corrected Wilcoxon test: *p* < 0.00071). The effect of lower PAC-ESC scores during HFS for Aβ-animals **(B)**, clearly results from lower envelope-to-signal correlations at high gamma center-frequencies. This can also observed by eye if one compares the PAC-ESC scores of control and Aβ-group during single epochs of HFS application **(C,D)**. In addition, the pooled data obtained during single epochs of HFS application **(E)** reflect an increase in envelope-to-signal correlations relative to time in control animals. This cannot be observed for the Aβ-group. Graphs in **(B,D)** are expressed as Mean ± SEM.

In addition to cross-frequency coupling in the form of phase synchrony (Tass et al., 1998), phase-to-amplitude coupling (PAC) can also occur, whereby the phase of a lower-frequency rhythm modulates the amplitude of a higher-frequency oscillation (Onslow et al., 2011). PAC has already been described in the CA1 region, where gamma frequency exhibits cyclic fluctuations that are concurrent with changes in the theta phase (Bragin et al., 1995). To determine if the profile of changes in spectral power and trough-to-peak amplitudes in Aβ-treated animals also extends to PAC, we investigated PAC by verifying the envelope-to-signal correlation (ESC). The mean PAC-ESC scores over time for the control and Aβ-group (Figures 6A,B) did not reveal obvious differences in PAC-ESC coupling scores, as a consequence of either Aβ-treatment or HFS. However, PAC-ESC coupling occurred predominantly between low frequencies in the range of theta, alpha and beta bands, as well as high gamma envelopes in both control and Aβ-treated animals (see also Figure 2F). To determine the possible effects of Aβ on PAC-ESC coupling in range of theta-and gamma oscillations, we extracted the PAC-ESC scores for these frequencies from the entire PAC-ESC response for control and Aβ-group (Figure 7). A comparison of the relative changes in the pooled PAC-ESC scores over time for control and Aβ-treated animals (Figure 7B) revealed a significantly lower envelope-to-signal correlation for theta- and gamma oscillations for the Aβ-group, compared to the control group at all time-points measured.

This observation for the pooled data is however undermined by the fact that the baselines of the control and Aβ-groups (Figure 7, time point −100 s) differ significantly from each other. However, we did not observe significantly different baselines for field potentials (Figure 1), relative power data (Figure 3) or relative amplitudes (Figures 4, 5), so it is unlikely that the difference in baselines seen here was induced by factors unrelated to the Aβ-treatment, or the chosen time-windows for EEG analysis. To assess this, we examined changes in the relative PAC-ESC score of the single binned gamma oscillation envelopes (single center frequencies) to the theta signal and compared them for control and Aβ-group (Figure 7A). Only the center frequencies at 38 Hz (±2 Hz) differ in the baseline of control and Aβ-treated animals. This might affect the pooled data (Figure 7B) and may explain the significant difference in baselines between control and Aβ-treated animals.

The main effects of Aβ-treatment during HFS appear at PAC-ESC scores for high gamma frequencies to the theta signal, however (Figure 7A). The increase in envelope-to-signal correlations of the gamma signal envelopes at high center frequencies to the theta signal amplitudes seem to contribute to the higher PAC-ESC scores in the pooled data during HFS for control animals (Figure 7B, time point 0). The opposite effect can be observed for the Aβ-treated animals. Closer scrutiny of the change in PAC-ESC scores at the time-period of HFS induction (Figures 7C,D) reveals that, with ongoing HFS stimulation, the PAC-ESC scores in control animals are higher for fast gamma envelopes and theta amplitudes (Figure 7C). In contrast, the PAC-ESC scores for fast gamma envelopes and theta signal amplitudes are lowered during ongoing HFS stimulation. This can also be observed by comparison of the pooled PAC-ESC scores for control and Aβ-treated animals (Figure 7E). In general this means that Aβ-treatment lowers the probability that high gamma envelopes match to the shape of the theta signal amplitudes.

Evidence that relative power levels and the PAC-ESC scores are related on a functional level in from control animals can be seen by a comparison of the relative theta power levels during HFS (Figure 3B, second graph) and relative gamma power levels (Figure 3B, bottom graph) with the PAC-ESC scores at the same time windows (Figure 7E). A Friedman-Test revealed that a significant relationship exists between relative theta and gamma power and relative PAC-ESC scores during HFS for control animals only (${\text{χ}}_{\left(\text{9}\right)}^{\text{2}}$ = 20.6727, *p* = 0.01419) but not for the Aβ-treated animals (${\text{χ}}_{\left(\text{9}\right)}^{\text{2}}$ = 9.1091, *p* = 0.4273). However, a comparison of relative PAC-ESC scores of control animals to changes in relative theta power alone revealed no significant relationship during HFS (Friedman-Test: ${\text{χ}}_{\left(\text{9}\right)}^{\text{2}}$ = 14.9455, *p* = 0.09245). The same is true for a comparison of PAC-ESC scores to relative gamma power during HFS in control animals (Friedman-Test: ${\text{χ}}_{\left(\text{9}\right)}^{\text{2}}$ = 14.2909, *p* = 0.1123). This can also be observed for Aβ-treated animals (PAC-ESC scores/relative theta power: (${\text{χ}}_{\left(\text{9}\right)}^{\text{2}}$ = 9.7091, *p* = 0.3745); PAC-ESC scores/relative gamma power: (${\text{χ}}_{\left(\text{9}\right)}^{\text{2}}$ = 9.2727, *p* = 0.4125)). A comparison of relative theta and gamma power alone, also revealed no significant relationship for control animals (${\text{χ}}_{\left(\text{9}\right)}^{\text{2}}$ = 15.2727, *p* = 0.08371), or for Aβ-treated animals ${\text{χ}}_{\left(\text{9}\right)}^{\text{2}}$ = 8.1818, *p* = 0.5159). Nonetheless, based on the *p*-values of these tests one can see that the relationship between PAC-ESC scores and relative theta-and gamma power is tighter in control animals than in the Aβ-group. The absence of a significant connection between envelope to signal correlations and relative power values in Aβ-treated animals may thus contribute to the failure in expression of LTP. Of course by binning the gamma frequency into 5 Hz bands we can only approximate the effect of Aβ on the sub-frequencies of the gamma oscillation and their relationship to the theta signal amplitudes. The true frequency bands might show unequal and temporally changing bandwidths with non-integer band widths. Nonetheless the results for theta-gamma PAC-ESC scores show that Aβ-treatment affects the ability of the DG network to mediate coupling between the gamma envelopes and the theta signal amplitudes. This in turn suggests that Aβ-treatment causes changes in cross-frequency coupling of neuronal activity, that may consequently contribute to LTP failure.

## Discussion

In this study, we report that a single, acute application of Aβ(1–42) impairs LTP in the dentate gyrus that normally persists for over 24 h in freely behaving rats. Intrahippocampal EEG analysis before, during and immediately after HFS to induce LTP, revealed that theta and gamma oscillations are altered in Aβ(1–42)-treated animals compared to controls. Alterations in delta, alpha and beta oscillations are also evident. These data indicate that the deficits in hippocampal LTP that occur following Aβ-treatment are accompanied by disturbances in hippocampal information transfer via neuronal oscillations. Neuronal oscillations typically accompany information processing in the hippocampus (Buzsáki and Chrobak, 1995; Jensen and Lisman, 1996; Lisman, 2005; Sullivan et al., 2011), whereby synaptic plasticity is believed to comprise the cellular basis for learning and memory (Kemp and Manahan-Vaughan, 2007). In the hippocampus, the theta activity (4–10 Hz in rats) reaches its highest power during novel spatial exploration or REM sleep (Buzsáki, 2002). Gamma oscillations (30–100 Hz in rats) are closely associated with theta activity, and exhibit their highest amplitudes in the DG of the hippocampus (Csicsvari et al., 2003; Bartos et al., 2007). They are also believed to enable binding features of sensory signals (Singer, 1993) and contribute to consciousness (Llinás et al., 1998) and long-term information storage (de Almeida et al., 2007). Whether HFS will successfully induce LTP, result in STP, or fails to elicit a change in synaptic strength can be predicted by the relative change in theta and gamma activity during and after HFS (Bikbaev and Manahan-Vaughan, 2007, 2008). In the present study, HFS applied to control animals, which in all cases resulted in LTP (>24 h), resulted in a very similar pattern that was not evident in Aβ–treated animals. Along with failed LTP induction, and disrupted theta power and gamma amplitudes during theta cycles recorded in association with HFS, we observed that the coupling between gamma envelopes and theta signal amplitudes is not significantly connected to the changes in relative theta and gamma power during HFS in Aβ-treated animals, as is the case in control animals.

Our findings with regard to healthy animals support previous reports (Bikbaev and Manahan-Vaughan, 2007) that showed that successful induction of LTP by HFS is accompanied by a transient suppression of the relative theta power and peak-to-slope amplitudes at sub-frequencies in the range of the theta band. In contrast to this previous study however, the Aβ-treated animals examined in the present study were not *naive* in terms of LTP induction. In the current study, animals served as their own controls, whereby LTP was first assessed prior to Aβ-treatment. This was essential, as we needed to be sure that the animal that received Aβ were able to express robust LTP under control circumstances. However, at least 7 days elapsed before we assessed the effects of Aβ-treatment on LTP after the initial control LTP experiment. The input-output curve (stimulus-response relationship) that was obtained prior to commencing each experiment was compared for each individual animal to confirm that LTP was no longer present. Thus, although we would think it unlikely that there were residual effects, we cannot entirely exclude that the underlying network was trained by the control HFS-induction, and this might affect the EEG power levels we observed when LTP was assessed in the presence of Aβ. The theta-gamma power patterns expressed by the Aβ-treated animals following HFS, were completely distinct from those patterns observed in healthy animals that expressed LTP, short-term plasticity or failure of LTP after HFS (Bikbaev and Manahan-Vaughan, 2007, 2008), whereas the responses of the control animals to HFS were equivalent to what we previously observed (Bikbaev and Manahan-Vaughan, 2007, 2008). In addition we observed changes in cross-frequency coupling.

After acute treatment with oligomeric Aβ(1–42), LTP was impaired in animals that had previously expressed LTP under control conditions. In particular, the maintenance of LTP was affected. LTP maintenance *in vivo* critically depends on the activation of metabotropic glutamate (mGlu) receptors (Mukherjee and Manahan-Vaughan, 2013). However, mGlu5 is not only required for persistent LTP but also for support of hippocampal neuronal oscillations related to LTP induction (Bikbaev et al., 2008). Interestingly, the Aβ-oligomer impairs mGlu5 function (Renner et al., 2010) and mGlu5 mediates enhancement of LTD that are triggered by Aβ(Hu et al., 2014). The binding of Aβ to mGlu5 is believed to be mediated by the cellular prion protein (PrP*^c^*) in connection with Fyn-tyrosine kinase (Um et al., 2013). Thus, the failure of LTP might be linked to Aβ-mediated disruptions of mGlu5 receptor function. Other factors could contribute to the failure of LTP mediated by acute Aβ-injection. The abnormal folding of the Aβ(1–42)-oligomer (Yun et al., 2007) facilitates its binding to metal ions leading to its high toxicity (Faller and Hureau, 2012). Aggregation of Aβ with zinc and copper may also lead to over-activation of the N-methyl-D-aspartate receptor (NMDAR) and thereby to an enhanced Ca^2+^ influx in the presence of glutamate. This may change excitability levels and alter the ability of the synapse to sustain LTP. STP was induced by HFS in Aβ–treated animals, but the profile of EEG responses was not equivalent to those seen when HFS resulted in STP in healthy animals (Bikbaev and Manahan-Vaughan, 2007). This suggests that additional mechanisms mediate the disruption of theta and gamma power, that in turn are associated with impaired LTP in Aβ–treated animals.

When we examined gamma oscillations that were nested within theta cycles (Penny et al., 2008; Tort et al., 2010), we detected higher relative gamma amplitudes during HFS in Aβ-treated animals compared to controls. This kind of response can be expected if theta-driving pyramidal neurons show higher activity, because they are physiological connected to the gamma oscillation-inducing parvalbumin-positive interneurons (Fuchs et al., 2007; Buzsáki and Wang, 2012). Effects during HFS were significant for theta cycles in the frequency ranges from 5–10 Hz, which would include both atropine-sensitive (6–7 Hz) and atropine-insensitive theta (~8 Hz) (Kramis et al., 1975; Whishaw et al., 1991). These differences in Aβ-treated animals may result from impaired inhibition of theta oscillations, and thereby a stronger activation of GABA_A_-ergic interneurons, or a lower activation of the interneurons themselves, because of lesser GABA_A_-receptor activation (Buzsáki and Wang, 2012). Interestingly, the processing of novel visual information by the visual cortex is coupled to theta activity in the dentate gyrus *in vivo* that occurs in the 8 Hz range (Tsanov and Manahan-Vaughan, 2009). Thus, the specific effect of Aβ-treatment on 8 Hz theta peak-to-slope amplitudes following HFS may reflect a functional disruption in the dentate gyrus.

Gamma oscillations originate locally from parvalbumin-positive interneurons that are interlinked via gap junctions (Buzsáki and Draguhn, 2004). As they are directly affected by changes in the theta oscillations, the changes in relative gamma power and mean gamma amplitudes during theta cycles that we observed in Aβ-treated animals may have derived from disruptions in the ability of the DG to regulate theta oscillatory behavior. Bearing in mind that changes in theta will affect gamma oscillations, we propose that the main effects of Aβ are on the theta oscillations.

Delta, theta and alpha bands originate outside the hippocampus. Delta oscillations originate in thalamocortical cells (McCormick and Bal, 1997) and reach the hippocampus via the nucleus reuniens projection of the thalamus to the CA1 region (Zhang et al., 2012). Slow neural oscillations are not only associated with deep-sleep states, but may be relevant for selective attention (Schroeder and Lakatos, 2009). In our study we found that the relative delta power is enhanced and responses exhibit an unstable pattern during HFS application in Aβ-treated animals. This might contribute to disruptions in sensory information processing in the DG. In addition to delta oscillations, alpha oscillations also originate from the thalamus (Hughes and Crunelli, 2005). Alpha oscillations exhibit cross-frequency phase coupling with gamma and beta oscillations during working memory and perception (von Stein et al., 2000) and have been proposed to underlie working memory (Chik, 2013). We observed that the relative alpha power reached extremely high values during HFS and afterwards, in Aβ-treated animals. This contrasts with the reduced alpha bursts that have been reported at the temporal-parietal cortex in AD patients (Montez et al., 2009). However cortical and hippocampal patterns of network disruptions might differ in deflection. Our results support that disruptions in the alpha band occur in AD. Endogenous application of Aβ results in impaired working memory in rats (Pearson-Leary and McNay, 2012). This may derive in part from the alterations in alpha power detected in Aβ-treated animals in our study. A relationship between learning and beta oscillations in rodents (Berke et al., 2008) and in cats (Múnera et al., 2001) has also been shown, where it was reported that the beta power increases during exploration of a new environment, but decays if the environment is familiar. Interestingly, beta oscillatory activity in hippocampal CA1 pyramidal cells was shown to be time-locked to the paired presentation of conditioned and unconditioned stimuli, suggesting that beta oscillations operate as a temporal permissive window to facilitate hippocampal output information streams to higher levels of neuronal processing (Múnera et al., 2001). Our data shows that the relative beta power levels are raised during and after HFS in Aβ-treated rodents. Abnormal resting-states in EEG rhythms at the cortical level in AD patients have been reported (Babiloni et al., 2010; Hsiao et al., 2013). Furthermore, reductions in cortical power levels of the alpha and beta bands and increases in theta and delta activity were observed. Synchronized activity in different cortical sub-networks that is mediated and reflected by beta oscillations has been shown in the rat and human frontal cortex (van Aerde et al., 2009). We observed that relative beta power levels are increased during HFS of Aβ-treated animals and remain significant higher during the subsequent 300 s compared to controls. Therefore, the cognitive processes underlying beta band synchronization might be disrupted in Aβ-group animals.

A suppression of theta power and elevation in gamma power associated with HFS predict for the probability of successful hippocampal LTP induction in the dentate gyrus of naive rats (Bikbaev and Manahan-Vaughan, 2007, 2008). A tight relationship between gamma oscillations and theta oscillations exists in the hippocampus, because of their interconnection on the physiological level (Vida et al., 2006; Fuchs et al., 2007; Lubenov and Siapas, 2009). Theta oscillations in the hippocampus have different sources, such as the CA3 (Cobb et al., 2003), and CA1 regions (Gillies et al., 2002), but also are transferred to the hippocampus from the medial septum (Dragoi et al., 1999). The dentate gyrus is unlikely to serve as a theta pacemaker (Kowalczyk et al., 2009). The theta oscillations within the hippocampus are generated by persistent synchronous cholinergic-based firing of pyramidal neurons that are periodically inhibited by GABA_B_-receptor activation (Stewart and Fox, 1990; Buzsáki, 2002). Tightly connected to these pyramidal neurons, inhibitory GABA_A_-dependent interneurons are activated and generate high-frequency gamma oscillations (Wallenstein and Hasselmo, 1997; Leung, 1998). Hyperpolarizing shunting inhibition from the gamma oscillations to the pyramidal cells (Lamsa and Taira, 2003) may in turn regulate this recurrent inhibition-based network (Vida et al., 2006). We could observe that the relative theta power during HFS in healthy animals is temporally suppressed, but not throughout the whole HFS time window (Figure 3B, second graph from top).

Enhanced GABA_B_-receptor activity might first protect the underlying network from over-activation during HFS. In line with this, we observed that during HFS the relative theta and gamma power rises and shows enhanced probability of envelope-to-signal correlations (Figure 7E). Correlations between the theta signal and gamma envelopes may raise the probability of LTP expression. Thus, the lowered PAC-ESC scores that we observed for gamma envelopes relative to theta signal amplitudes in Aβ-treated animals, may result indirectly from impaired GABA_B_-receptor activation. Taking into account the contribution of theta phase-reset in the regulation of the probability of LTP occurrence (McCartney et al., 2004), and the importance of gamma-induced shunting inhibition of theta power, the impaired ability of the theta-gamma frequency bands to express envelope-to-signal correlations observed in our study might not only accompany the failure in LTP, but may also actively influence the underlying pathological process. From the perspective of sensory information processing, the theta-gamma impairments seen during and after HFS in Aβ-treated animals might also reflect a failure in shift of the system to a new dynamic state (Buzsáki, 2006). In the CA3 region of urethane-anesthetized young rats, cycle-by-cycle fluctuations in gamma amplitude reflect changes in the dynamic regulation of synaptic excitation and inhibition (Atallah and Scanziani, 2009). Furthermore, the precise phase of gamma oscillations can determine whether or not activity is effectively transmitted between cortical areas (Womelsdorf et al., 2007). Behavioral state exerts a very potent influence on gamma oscillatory activity. To ensure that our comparisons of oscillatory activity were as comparable as possible across animals, we ensured that all animals were stationary, resting and had their eyes open prior to during and immediately after HFS. The patterns in relative theta and gamma power levels we observe for Aβ-treated animals are not comparable to the patterns expressed in healthy animals for successful expression of LTP, STP of failure in potentiation. This observation goes along with the lower appearance of envelope-to-signal correlations during HFS in Aβ-group animals.

## Conclusions

Taken together these results indicate that changes in neuronal oscillations occur as a result of for Aβ-treatment that are most particularly evident with regard to theta and gamma activity. Disruptions of the theta-gamma relationship occurs during processes that should lead to hippocampal LTP will have profound consequences not only for the stability and successful induction of LTP, but also for cognitive processes that depend on LTP. We propose that this is part of a pathological pattern that may underlie cognitive deficits in AD.

## Conflict of Interest Statement

The authors declare that the research was conducted in the absence of any commercial or financial relationships that could be construed as a potential conflict of interest.

## References

[B1] Adaya-Villanueva A. Ordaz B. Balleza-Tapia H. Márquez-Ramos A. Peña-Ortega F. (2010). Beta-like hippocampal network activity is differentially affected by amyloid beta peptides. Peptides 31, 1761–1766. 10.1016/j.peptides.2010.06.003 20558221

[B4] Atallah B. V. Scanziani M. (2009). Instantaneous modulation of gamma oscillation frequency by balancing excitation with inhibition. Neuron 62, 566–577. 10.1016/j.neuron.2009.04.027 19477157 PMC2702525

[B5] Babiloni C. Lizio R. Vecchio F. Frisoni G. B. Pievani M. Geroldi C.. (2010). Reactivity of cortical alpha rhythms to eye opening in mild cognitive impairment and Alzheimer’s disease: an EEG study. J. Alzheimers Dis. 22, 1047–1064. 10.3233/JAD-2010-100798 20930306

[B6] Babri S. Amani M. Mohaddes G. Alihemmati A. Ebrahimi H. (2012). Effect of aggregated β-Amyloid (1–42) on synaptic plasticity of hippocampal dentate gyrus granule cells *in vivo*. Bioimpacts 2, 189–194. 10.5681/bi.2012.022 23678459 PMC3648935

[B7] Barghorn S. Nimmrich V. Striebinger A. Krantz C. Keller P. Janson B.. (2005). Globular amyloid beta-peptide oligomer - a homogenous and stable neuropathological protein in Alzheimer’s disease. J. Neurochem. 95, 834–847. 10.1111/j.1471-4159.2005.03407.x 16135089

[B8] Bartos M. Vida I. Jonas P. (2007). Synaptic mechanisms of synchronized gamma oscillations in inhibitory interneuron networks. Nat. Rev. Neurosci. 8, 45–56. 10.1038/nrn2044 17180162

[B9] Battaglia F. P. Benchenane K. Sirota A. Pennartz C. M. Wiener S. I. (2011). The hippocampus: hub of brain network communication for memory. Trends Cogn. Sci. 15, 310–318. 10.1016/j.tics.2011.05.008 21696996

[B10] Berke J. D. Hetrick V. Breck J. Greene R. W. (2008). Transient 23–30 Hz oscillations in mouse hippocampus during exploration of novel environments. Hippocampus 18, 519–529. 10.1002/hipo.20435 18398852

[B11] Bikbaev A. Manahan-Vaughan D. (2007). Hippocampal network activity is transiently altered by induction of long-term potentiation in the dentate gyrus of freely behaving rats. Front. Behav. Neurosci. 1:7. 10.3389/neuro.08.007.2007 18958189 PMC2525854

[B12] Bikbaev A. Manahan-Vaughan D. (2008). Relationship of hippocampal theta and gamma oscillations to potentiation of synaptic transmission. Front. Neurosci. 2, 56–63. 10.3389/neuro.01.010.2008 18982107 PMC2570077

[B13] Bikbaev A. Neyman S. Ngomba R. T. Conn J. Nicoletti F. Manahan-Vaughan D.. (2008). MGluR5 mediates the interaction between late-LTP, network activity and learning. PLoS One 3:e2155. 10.1371/journal.pone.0002155 18478073 PMC2364645

[B15] Bland B. H. (1986). The physiology and pharmacology of hippocampal formation theta rhythms. Prog. Neurobiol. 26, 1–54. 10.1016/0301-0082(86)90019-5 2870537

[B16] Bozsó Z. Penke B. Simon D. Laczkó I. Juhász G. Szegedi V.. (2010). Controlled *in situ* preparation of a beta(1–42) oligomers from the isopeptide “iso-A beta(1–42)”, physicochemical and biological characterization. Peptides 31, 248–256. 10.1016/j.peptides.2009.12.001 19995586

[B113] Bragin A. Jandó G. Nádasdy Z. Hetke J. Wise K. G. Buzsáki G. (1995). Gamma (40-100 Hz) oscillation in the hippocampus of the behaving rat. J. Neurosci. 15, 47–60. 7823151 10.1523/JNEUROSCI.15-01-00047.1995 PMC6578273

[B17] Bruns A. Eckhorn R. (2004). Task-related coupling from high- to low-frequency signals among visual cortical areas in human subdural recordings. Int. J. Psychophysiol. 51, 97–116. 10.1016/j.ijpsycho.2003.07.001 14693360

[B18] Buzsáki G. (2002). Theta oscillations in the hippocampus. Neuron 33, 325–340. 10.1016/s0896-6273(02)00586-x 11832222

[B19] Buzsáki G. Chrobak J. J. (1995). Temporal structure in spatially organized neuronal ensembles: a role for interneuronal networks. Curr. Opin. Neurobiol. 5, 504–510. 10.1016/0959-4388(95)80012-3 7488853

[B21] Buzsáki G. (2006). Rhythms of the Brain. New York: Oxford University Press.

[B20] Buzsáki G. Draguhn A. (2004). Neuronal oscillations in cortical networks. Science 304, 1926–1929. 10.1126/science.1099745 15218136

[B22] Buzsáki G. Wang X. (2012). Mechanisms of gamma oscillations. Annu. Rev. Neurosci. 35, 203–225. 10.1146/annurev-neuro-062111-150444 22443509 PMC4049541

[B23] Cairns N. J. Chadwick A. Luthert P. J. Lantos P. L. (1991). beta-Amyloid protein load is relatively uniform throughout neocortex and hippocampus in elderly Alzheimer’s disease patients. Neurosci. Lett. 129, 115–118. 10.1016/0304-3940(91)90733-a 1922960

[B24] Canolty R. T. Edwards E. Dalal S. S. Soltani M. Nagarajan S. S. Kirsch H. E.. (2006). High gamma power is phase-locked to theta oscillations in human neocortex. Science 313, 1626–1628. 10.1126/science.1128115 16973878 PMC2628289

[B25] Chen Q. S. Kagan B. L. Hirakura Y. Xie C. W. (2000). Impairment of hippocampal long-term potentiation by Alzheimer amyloid beta-peptides. J. Neurosci. Res. 60, 65–72. 10.1002/(sici)1097-4547(20000401)60:1<65::aid-jnr7>3.0.co;2-q 10723069

[B26] Cheng S. (2013). The CRISP theory of hippocampal function in episodic memory. Front. Neural Circuits. 7:88. 10.3389/fncir.2013.00088 23653597 PMC3644677

[B27] Chik D. (2013). Theta-alpha cross-frequency synchronization facilitates working memory control - a modeling study. Springerplus 2:14. 10.1186/2193-1801-2-14 23440395 PMC3574971

[B28] Cleary J. P. Walsh D. M. Hofmeister J. J. Shankar G. M. Kuskowski M. A. Selkoe D. J.. (2005). Natural oligomers of the amyloid-beta protein specifically disrupt cognitive function. Nat. Neurosci. 8, 79–84. 10.1038/nn1372 15608634

[B29] Cobb S. R. Larkman P. M. Bulters D. O. Oliver L. Gill C. H. Davies C. H. (2003). Activation of Ih is necessary for patterning of mGluR and mAChR induced network activity in the hippocampal CA3 region. Neuropharmacology 44, 293–303. 10.1016/s0028-3908(02)00405-7 12604089

[B30] Csicsvari J. Jamieson B. Wise K. D. Buzsáki G. (2003). Mechanisms of gamma oscillations in the hippocampus of the behaving rat. Neuron 37, 311–322. 10.1016/s0896-6273(02)01169-8 12546825

[B31] Cullen W. K. Suh Y. H. Anwyl R. Rowan M. J. (1997). Block of LTP in rat hippocampus *in vivo* by beta-amyloid precursor protein fragments. Neuroreport 8, 3213–3217. 10.1097/00001756-199710200-00006 9351645

[B3] de Almeida L. Idiart M. Lisman J. E. (2007). Memory retrieval time and memory capacity of the CA3 network: role of gamma frequency oscillations. Learn. Mem. 14, 795–806. 10.1101/lm.730207 18007022 PMC2080581

[B32] Dees R. L. Kesner R. P. (2013). The role of the dorsal dentate gyrus in object and object-context recognition. Neurobiol. Learn. Mem. 106, 112–117. 10.1016/j.nlm.2013.07.013 23880567

[B33] Dragoi G. Buzsáki G. (2006). Temporal encoding of place sequences by hippocampal cell assemblies. Neuron 50, 145–157. 10.1016/j.neuron.2006.02.023 16600862

[B34] Dragoi G. Carpi D. Recce M. Csicsvari J. Buzsáki G. (1999). Interactions between hippocampus and medial septum during sharp waves and theta oscillation in the behaving rat. J. Neurosci. 19, 6191–6199. 10407055 10.1523/JNEUROSCI.19-14-06191.1999 PMC6783073

[B35] Faller P. Hureau C. (2012). A bioinorganic view of Alzheimer’s disease: when misplaced metal ions (re)direct the electrons to the wrong target. Chemistry 18, 15910–15920. 10.1002/chem.201202697 23180511

[B37] Fuchs E. C. Zivkovic A. R. Cunningham M. O. Middleton S. L. Fiona E. N. Bannerman D. M.. (2007). Recruitment of parvalbumin-positive interneurons determines hippocampal function and associated behavior. Neuron 53, 591–604. 10.1016/j.neuron.2007.01.031 17296559

[B38] Geng Y. Li C. Liu J. Xing G. Zhou L. Dong M.. (2010). Beta-asarone improves cognitive function by suppressing neuronal apoptosis in the beta-amyloid hippocampus injection rats. Biol. Pharm. Bull. 33, 836–843. 10.1248/bpb.33.836 20460763

[B39] Gillies M. J. Traub R. D. LeBeau F. E. N. Davies C. H. Gloveli T. Buhl E. H.. (2002). A model of atropine-resistant theta oscillations in rat hippocampal area CA1. J. Physiol. 543, 779–793. 10.1113/jphysiol.2002.024588 12231638 PMC2290530

[B41] Gray C. M. König P. Engel A. K. Singer W. (1989). Oscillatory responses in cat visual cortex exhibit inter-columnar synchronization which reflects global stimulus properties. Nature 338, 334–337. 10.1038/338334a0 2922061

[B42] Gruart A. López-Ramos J. C. Muñoz M. D. Delgado-García J. M. (2008). Aged wild-type and APP, PS1 and APP + PS1 mice present similar deficits in associative learning and synaptic plasticity independent of amyloid load. Neurobiol. Dis. 30, 439–450. 10.1016/j.nbd.2008.03.001 18442916

[B44] Habib D. Tsui C. K. Y. Rosen L. G. Dringenberg H. C. (2013). Occlusion of low-frequency-induced, heterosynaptic long-term potentiation in the rat hippocampus *in vivo* following spatial training. Cereb. Cortex 24, 3090–3096. 10.1093/cercor/bht174 23825318

[B45] Hansen N. Manahan-Vaughan D. (2014). Locus coeruleus stimulation facilitates long-term depression in the dentate gyrus that requires activation of β-adrenergic receptors. Cereb. Cortex [Epub ahead of print]. 10.1093/cercor/bht429 24464942 PMC4459289

[B46] Hardy J. Selkoe D. J. (2002). The amyloid hypothesis of Alzheimer’s disease: progress and problems on the road to therapeutics. Science 297, 353–356. 10.1126/science.1072994 12130773

[B48] Hsiao F. Wang Y. Yan S. Chen W. Lin Y. (2013). Altered oscillation and synchronization of default-mode network activity in mild Alzheimer’s disease compared to mild cognitive impairment: an electrophysiological study. PLoS One 8:e68792. 10.1371/journal.pone.0068792 23874766 PMC3708894

[B49] Hu N. W. Nicoll A. J. Zhang D. Mably A. J. O’Malley T. Purro S. A.. (2014). mGlu5 receptors and cellular prion protein mediate amyloid-β-facilitated synaptic long-term depression *in vivo*. Nat. Commun. 5:3374. 10.1038/ncomms4374 24594908 PMC4354159

[B50] Hughes S. W. Crunelli V. (2005). Thalamic mechanisms of EEG alpha rhythms and their pathological implications. Neuroscientist 11, 357–372. 10.1177/1073858405277450 16061522

[B51] Jensen O. Lisman J. E. (1996). Hippocampal CA3 region predicts memory sequences: accounting for the phase precession of place cells. Learn. Mem. 3, 279–287. 10.1101/lm.3.2-3.279 10456097

[B52] Karran E. Mercken M. De Strooper B. (2011). The amyloid cascade hypothesis for Alzheimer’s disease: an appraisal for the development of therapeutics. Nat. Rev. Drug Discov. 10, 698–712. 10.1038/nrd3505 21852788

[B53] Kemp A. Manahan-Vaughan D. (2007). Hippocampal long-term depression: master or minion in declarative memory processes? Trends Neurosci. 30, 111–118. 10.1016/j.tins.2007.01.002 17234277

[B54] Kemp A. Manahan-Vaughan D. (2008). β-adrenoreceptors comprise a critical element in learning-facilitated long-term plasticity. Cereb. Cortex 18, 1326–1334. 10.1093/cercor/bhm164 17906333

[B56] Klyubin I. Walsh D. M. Cullen W. K. Fadeeva J. V. Anwyl R. Selkoe D. J.. (2004). Soluble Arctic amyloid beta protein inhibits hippocampal long-term potentiation *in vivo*. Eur. J. Neurosci. 19, 2839–2846. 10.1111/j.1460-9568.2004.03389.x 15147317

[B57] Kowalczyk T. Gołebiewski H. Konopacki J. (2009). Is the dentate gyrus an independent generator of *in vitro* recorded theta rhythm? Brain Res. Bull. 80, 139–146. 10.1016/j.brainresbull.2009.07.003 19615430

[B58] Kramis R. Vanderwolf C. H. Bland B. H. (1975). Two types of hippocampal rhythmical slow activity in both the rabbit and the rat: relations to behavior and effects of atropine, diethyl ether, urethane and pentobarbital. Exp. Neurol. 49, 58–85. 10.1016/0014-4886(75)90195-8 1183532

[B59] Lambert M. P. Barlow A. K. Chromy B. A. Edwards C. Freed R. Liosatos M.. (1998). Diffusible, nonfibrillar ligands derived from Abeta1–42 are potent central nervous system neurotoxins. Proc. Natl. Acad. Sci. U S A 95, 6448–6453. 10.1073/pnas.95.11.6448 9600986 PMC27787

[B60] Lamsa K. Taira T. (2003). Use-dependent shift from inhibitory to excitatory GABA_A_ receptor action in SP-O interneurons in the rat hippocampal CA3 area. J. Neurophysiol. 90, 1983–1995. 10.1152/jn.00060.2003 12750426

[B61] Leung L. S. (1998). Generation of theta and gamma rhythms in the hippocampus. Neurosci. Biobehav. Rev. 22, 275–290. 10.1016/s0149-7634(97)00014-6 9579318

[B62] Lisman J. (2005). The theta/gamma discrete phase code occuring during the hippocampal phase precession may be a more general brain coding scheme. Hippocampus 15, 913–922. 10.1002/hipo.20121 16161035

[B63] Lisman J. E. Idiart M. A. (1995). Storage of 7 +/− 2 short-term memories in oscillatory subcycles. Science 267, 1512–1515. 10.1126/science.7878473 7878473

[B64] Llinás R. Ribary U. Contreras D. Pedroarena C. (1998). The neuronal basis for consciousness. Philos. Trans. R. Soc. Lond. B Biol. Sci. 353, 1841–1849. 10.1098/rstb.1998.0336 9854256 PMC1692417

[B65] Lopes da Silva F. H. Witter M. P. Boeijinga P. H. Lohman A. H. (1990). Anatomic organization and physiology of the limbic cortex. Physiol. Rev. 70, 453–511. 2181500 10.1152/physrev.1990.70.2.453

[B66] Lubenov E. V. Siapas A. G. (2009). Hippocampal theta oscillations are travelling waves. Nature 459, 534–539. 10.1038/nature08010 19489117

[B67] Lyons A. Griffin R. J. Costelloe C. E. Clarke R. M. Lynch M. A. (2007). IL-4 attenuates the neuroinflammation induced by amyloid-beta *in vivo* and *in vitro*. J. Neurochem. 101, 771–781. 10.1111/j.1471-4159.2006.04370.x 17250684

[B68] McCartney H. Johnson A. D. Weil Z. M. Givens B. (2004). Theta reset produces optimal conditions for long-term potentiation. Hippocampus 14, 684–687. 10.1002/hipo.20019 15318327

[B69] McCormick D. A. Bal T. (1997). Sleep and arousal: thalamocortical mechanisms. Annu. Rev. Neurosci. 20, 185–215. 10.1146/annurev.neuro.20.1.185 9056712

[B70] Montez T. Poil S. Jones B. F. Manshanden I. Verbunt J. P. A. van Dijk B. W.. (2009). Altered temporal correlations in parietal alpha and prefrontal theta oscillations in early-stage Alzheimer disease. Proc. Natl. Acad. Sci. U S A 106, 1614–1619. 10.1073/pnas.0811699106 19164579 PMC2635782

[B71] Mukherjee S. Manahan-Vaughan D. (2013). Role of metabotropic glutamate receptors in persistent forms of hippocampal plasticity and learning. Neuropharmacology 66, 65–81. 10.1016/j.neuropharm.2012.06.005 22743159

[B72] Múnera A. Gruart A. Muñoz M. D. Fernández-Mas R. Delgado-García J. M. (2001). Hippocampal pyramidal cell activity encodes conditioned stimulus predictive value during classical conditioning in alert cats. J. Neurophysiol. 86, 2571–2582. 11698543 10.1152/jn.2001.86.5.2571

[B73] Naie K. Manahan-Vaughan D. (2004). Regulation by metabotropic glutamate receptor 5 of LTP in the dentate gyrus of freely moving rats: relevance for learning and memory formation. Cereb. Cortex 14, 189–198. 10.1093/cercor/bhg118 14704216

[B74] Ogomori K. Kitamoto T. Tateishi J. Sato Y. Suetsugu M. Abe M. (1989). Beta-protein amyloid is widely distributed in the central nervous system of patients with Alzheimer’s disease. Am. J. Pathol. 134, 243–251. 2464938 PMC1879581

[B75] Onslow A. C. E. Bogacz R. Jones M. W. (2011). Quantifying phase-amplitude coupling in neuronal network oscillations. Prog. Biophys. Mol. Biol. 105, 49–57. 10.1016/j.pbiomolbio.2010.09.007 20869387

[B77] Pearson-Leary J. McNay E. C. (2012). Intrahippocampal administration of amyloid-β(1–42) oligomers acutely impairs spatial working memory, insulin signaling and hippocampal metabolism. J. Alzheimers Dis. 30, 413–422. 10.3233/JAD-2012-112192 22430529 PMC5674792

[B78] Penny W. D. Duzel E. Miller K. J. Ojemann J. G. (2008). Testing for nested oscillation. J. Neurosci. Methods 174, 50–61. 10.1016/j.jneumeth.2008.06.035 18674562 PMC2675174

[B79] Pernía-Andrade A. J. Jonas P. (2014). Theta-gamma-modulated synaptic currents in hippocampal granule cells *in vivo* define a mechanism for network oscillations. Neuron 81, 140–152. 10.1016/j.neuron.2013.09.046 24333053 PMC3909463

[B80] Renner M. Lacor P. N. Velasco P. T. Xu J. Contractor A. Klein W. L.. (2010). Deleterious effects of amyloid beta oligomers acting as an extracellular scaffold for mGluR5. Neuron 66, 739–754. 10.1016/j.neuron.2010.04.029 20547131 PMC3111138

[B81] Sánchez-Alavez M. Chan S. L. Mattson M. P. Criado J. R. (2007). Electrophysiological and cerebrovascular effects of the alpha-secretase-derived form of amyloid precursor protein in young and middle-aged rats. Brain Res. 1131, 112–117. 10.1016/j.brainres.2006.10.074 17157827

[B82] Schroeder C. E. Lakatos P. (2009). Low-frequency neuronal oscillations as instruments of sensory selection. Trends Neurosci. 32, 9–18. 10.1016/j.tins.2008.09.012 19012975 PMC2990947

[B83] Selkoe D. J. (2002). Alzheimer’s disease is a synaptic failure. Science 298, 789–791. 10.1126/science.1074069 12399581

[B85] Singer W. (1993). Neuronal representations, assemblies and temporal coherence. Prog. Brain Res. 95, 461–474. 10.1016/s0079-6123(08)60388-x 8493353

[B88] Small D. H. McLean C. A. (1999). Alzheimer’s disease and the amyloid beta protein: what is the role of amyloid?. J. Neurochem. 73, 443–449. 10.1046/j.1471-4159.1999.0730443.x 10428038

[B89] Srivareerat M. Tran T. T. Alzoubi K. H. Alkadhi K. A. (2009). Chronic psychosocial stress exacerbates impairment of cognition and long-term potentiation in beta-amyloid rat model of Alzheimer’s disease. Biol. Psychiatry 65, 918–926. 10.1016/j.biopsych.2008.08.021 18849021

[B91] Stewart M. Fox S. E. (1990). Do septal neurons pace the hippocampal theta rhythm? Trends Neurosci. 13, 163–168. 10.1016/0166-2236(90)90040-h 1693232

[B92] Sullivan D. Csicsvari J. Mizuseki K. Montgomery S. Diba K. Buzsáki G. (2011). Relationships between hippocampal sharp waves, ripples and fast gamma oscillation: influence of dentate and entorhinal cortical activity. J. Neurosci. 31, 8605–8616. 10.1523/jneurosci.0294-11.2011 21653864 PMC3134187

[B93] Tass P. Rosenblum M. G. Weule J. Kurths J. Pikovsky A. Volkmann J. (1998). Detection of n:m phase locking from noisy data: application to magnetoencephalography. Phys. Rev. Lett. 81, 3291–3294 10.1103/physrevlett.81.3291

[B94] Tort A. Komorowski R. Eichenbaum H. Kopell N. (2010). Measuring phase-amplitude coupling between neuronal oscillations of different frequencies. J. Neurophysiol. 104, 1195–1210. 10.1152/jn.00106.2010 20463205 PMC2941206

[B95] Townsend M. Shankar G. M. Mehta T. Walsh D. M. Selkoe D. J. (2006). Effects of secreted oligomers of amyloid beta-protein on hippocampal synaptic plasticity: a potent role for trimers. J. Physiol. 572, 477–492. 10.1113/jphysiol.2005.103754 16469784 PMC1779683

[B96] Tsanov M. Manahan-Vaughan D. (2009). Visual cortex plasticity evokes excitatory alterations in the hippocampus. Front. Integr. Neurosci. 3:32. 10.3389/neuro.07.032.2009 19956399 PMC2786298

[B97] Tsuchiya K. Kosaka K. (1990). Neuropathological study of the amygdala in presenile Alzheimer’s disease. J. Neurol. Sci. 100, 165–173. 10.1016/0022-510x(90)90029-m 2089133

[B98] Um J. W. Kaufman A. C. Kostylev M. Heiss J. K. Stagi M. Takahashi H.. (2013). Metabotropic glutamate receptor 5 is a coreceptor for Alzheimer aβ oligomer bound to cellular prion protein. Neuron 79, 887–902. 10.1016/j.neuron.2013.06.036 24012003 PMC3768018

[B99] van Aerde K. I. Mann E. O. Canto C. B. Heistek T. S. Linkenkaer-Hansen K. Mulder A. B.. (2009). Flexible spike timing of layer 5 neurons during dynamic beta oscillation shifts in rat prefrontal cortex. J. Physiol. 587, 5177–5196. 10.1113/jphysiol.2009.178384 19752121 PMC2790257

[B100] Vanderwolf C. H. (1969). Hippocampal electrical activity and voluntary movement in the rat. Electroencephalogr. Clin. Neurophysiol. 26, 407–418. 10.1016/0013-4694(69)90092-3 4183562

[B101] Vertes R. P. Kocsis B. (1997). Brainstem-diencephalo-septohippocampal systems controlling the theta rhythm of the hippocampus. Neuroscience 81, 893–926. 10.1016/S0306-4522(97)00239-X 9330355

[B102] Vida I. Bartos M. Jonas P. (2006). Shunting inhibition improves robustness of gamma oscillations in hippocampal interneuron networks by homogenizing firing rates. Neuron 49, 107–117. 10.1016/j.neuron.2005.11.036 16387643

[B90] von Stein A. Chiang C. König P. (2000). Top-down processing mediated by interareal synchronization. Proc. Natl. Acad. Sci. U S A 97, 14748–14753. 10.1073/pnas.97.26.14748 11121074 PMC18990

[B104] Wallenstein G. V. Hasselmo M. E. (1997). GABAergic modulation of hippocampal population activity: sequence learning, place field development and the phase precession effect. J. Neurophysiol. 78, 393–408. 9242288 10.1152/jn.1997.78.1.393

[B105] Walsh D. M. Klyubin I. Fadeeva J. V. Cullen W. K. Anwyl R. Wolfe M. S.. (2002). Naturally secreted oligomers of amyloid beta protein potently inhibit hippocampal long-term potentiation *in vivo*. Nature 416, 535–539. 10.1038/416535a 11932745

[B107] Wang H. W. Pasternak J. F. Kuo H. Ristic H. Lambert M. P. Chromy B.. (2002). Soluble oligomers of beta amyloid (1–42) inhibit long-term potentiation but not long-term depression in rat dentate gyrus. Brain Res. 924, 133–140. 10.1016/S0006-8993(01)03058-X 11750898

[B108] Wang Q. W. Rowan M. J. Anwyl R. (2009). Inhibition of LTP by beta-amyloid is prevented by activation of beta2 adrenoceptors and stimulation of the cAMP/PKA signalling pathway. Neurobiol. Aging 30, 1608–1613. 10.1016/j.neurobiolaging.2007.12.004 18272254

[B109] Whishaw I. Q. Dyck R. Kolb B. (1991). Sparing of two types of hippocampal rhythmical slow activity (RSA, theta) in adult rats decorticated neonatally. Brain Res. Bull. 26, 425–427. 10.1016/0361-9230(91)90017-e 2049610

[B110] Womelsdorf T. Schoffelen J. M. Oostenveld R. Singer W. Desimone R. Engel A. K.. (2007). Modulation of neuronal interactions through neuronal synchronization. Science 316, 1609–1612. 10.1126/science.1139597 17569862

[B111] Yun S. Urbanc B. Cruz L. Bitan G. Teplow D. B. Stanley H. E. (2007). Role of electrostatic interactions in amyloid beta-protein (A beta) oligomer formation: a discrete molecular dynamics study. Biophys. J. 92, 4064–4077. 10.1529/biophysj.106.097766 17307823 PMC1868995

[B112] Zhang Y. Yoshida T. Katz D. B. Lisman J. E. (2012). NMDAR antagonist action in thalamus imposes delta oscillations on the hippocampus. J. Neurophysiol. 107, 3181–3189. 10.1152/jn.00072.2012 22423006 PMC3378362

